# Alternative splicing expands the clinical spectrum of NDUFS6-related mitochondrial disorders

**DOI:** 10.1016/j.gim.2024.101117

**Published:** 2024-06

**Authors:** Camila Armirola-Ricaurte, Noortje Zonnekein, Georgios Koutsis, Silvia Amor-Barris, Ana Lara Pelayo-Negro, Derek Atkinson, Stephanie Efthymiou, Valentina Turchetti, Argyris Dinopoulos, Antonio Garcia, Mert Karakaya, German Moris, Ayşe Ipek Polat, Uluç Yiş, Carmen Espinos, Liedewei Van de Vondel, Els De Vriendt, Georgia Karadima, Brunhilde Wirth, Michael Hanna, Henry Houlden, Jose Berciano, Albena Jordanova

**Affiliations:** 1 Molecular Neurogenomics group, VIB Center for Molecular Neurology, VIB, Antwerp, Belgium; 2 Molecular Neurogenomics group, Department of Biomedical Sciences, University of Antwerp, Antwerp, Belgium; 3 Neurogenetics Unit, 1st Department of Neurology, Eginitio Hospital, Medical School, National and Kapodistrian University of Athens, Athens, Greece; 4 University Hospital Marqués de Valdecilla (IFIMAV), University of Cantabria, Centro de Investigación Biomédica en Red de Enfermedades Neurodegenerativas (CIBERNED), Santander, Spain; 5 Department of Neuromuscular Disorders, UCL Institute of Neurology, Queen Square, London, United Kingdom; 6 3rd Department of Pediatrics, Attiko Hospital, Medical School, National and Kapodistrian University of Athens, Athens, Greece; 7 Service of Clinical Neurophysiology, University Hospital Marqués de Valdecilla, Centro de Investigación Biomédica en Red de Enfermedades Neurodegenerativas (CIBERNED), Santander, Spain; 8 Institute of Human Genetics, Center for Molecular Medicine Cologne, Center for Rare Diseases, University Hospital of Cologne, University of Cologne, Cologne, Germany; 9 Service of Neurology, University Hospital Central de Asturias, University of Oviedo, Oviedo, Spain; 10 Department of Pediatric Neurology, Dokuz Eylül University, Izmir, Turkey; 11 Rare Neurodegenerative Disease Laboratory, Centro de Investigación Príncipe Felipe (CIPF), CIBER on Rare Diseases (CIBERER), Valencia, Spain; 12 Translational Neurosciences, Faculty of Medicine and Health Sciences, University of Antwerp, Antwerp, Belgium; 13 Laboratory of Neuromuscular Pathology, Institute Born-Bunge, University of Antwerp, Antwerp, Belgium; 14 Department of Medical Chemistry and Biochemistry, Medical University-Sofia, Sofia, Bulgaria

**Keywords:** Charcot-Marie-Tooth, Mitochondrial disorders, NDUFS6, Peripheral neuropathy, Splicing

## Abstract

**Purpose:**

We describe 3 families with Charcot-Marie-Tooth neuropathy (CMT), harboring a homozygous *NDUFS6* NM_004553.6:c.309+5G>A variant previously linked to fatal Leigh syndrome. We aimed to characterize clinically and molecularly the newly identified patients and understand the mechanism underlying their milder phenotype.

**Methods:**

The patients underwent extensive clinical examinations. Exome sequencing was done in 4 affected individuals. The functional effect of the c.309+5G>A variant was investigated in patient-derived EBV-transformed lymphoblasts at the complementary DNA, protein, and mitochondrial level. Alternative splicing was evaluated using complementary DNA long-read sequencing.

**Results:**

All patients presented with early-onset, slowly progressive axonal CMT, and nystagmus; some exhibited additional central nervous system symptoms. The c.309+5G>A substitution caused the expression of aberrantly spliced transcripts and negligible levels of the canonical transcript. Immunoblotting showed reduced levels of mutant isoforms. No detectable defects in mitochondrial complex stability or bioenergetics were found.

**Conclusion:**

We expand the clinical spectrum of *NDUFS6*-related mitochondrial disorders to include axonal CMT, emphasizing the clinical and pathophysiologic overlap between these 2 clinical entities. This work demonstrates the critical role that alternative splicing may play in modulating the severity of a genetic disorder, emphasizing the need for careful consideration when interpreting splice variants and their implications on disease prognosis.

## Introduction

Complex I (CI), also known as reduced nicotinamide adenine dinucleotide-ubiquinone oxidoreductase, is the first of the 5 complexes of the oxidative phosphorylation system (OXPHOS) that generates an electrochemical gradient across the mitochondrial inner membrane to produce adenosine triphosphate (ATP).^1^, ^2^, ^3^ It is the largest component of the OXPHOS and consists of 44 subunits, 7 of them encoded by the mitochondrial DNA, and the remaining 37 encoded by nuclear DNA. These subunits work in concert to use the energy released by the electron transfer from nicotinamide adenine dinucleotide to ubiquinone to drive the proton translocation from the matrix to the intermembrane space.^1^^,^^2^^,^^4^^,^^5^ As a result, CI provides approximately 40% of the generated proton gradient required for ATP synthesis.^2^^,^^6^ Considering its size and functional contribution, CI deficiency can cause major energy metabolism impairment and represents the most common cause of OXPHOS disorders.^7^^,^^8^

Leigh syndrome is the most common clinical manifestation of CI deficiency.^9^^,^^10^ The disease is characterized by developmental regression, hypotonia, ataxia, movement impairment, and ophthalmological symptoms (ie, nystagmus and ophthalmoparesis). The symptoms typically develop by the second year of life, following an acute infection or illness. They are often accompanied by elevated serum or cerebrospinal fluid lactate and bilateral symmetrical lesions in the brainstem and basal ganglia. The disorder usually progresses in an episodic manner and results in death by 3 years of age because of respiratory or cardiac failure.^11^^,^^12^

NDUFS6 is a nuclear-encoded mitochondrial protein located in the matrix arm of CI. Despite being conserved from α-proteobacteria to man (Supplemental Figure 1), its exact function in CI activity remains unclear.^13^ Biallelic loss-of-function variants in *NDUFS6* (HGNC:7713) cause severe CI deficiency (MIM 618232) and Leigh syndrome.^3^^,^^14^, ^15^, ^16^, ^17^, ^18^, ^19^ A handful of patients have been described in the literature, and they all show severe lactic acidosis, hypotonia, feeding difficulties, and drowsiness. Most of them carry biallelic protein truncating variants and die within the first month of life because of respiratory failure.^14^^,^^15^ In contrast, a patient reported by Rouzier et al^3^ survived up to 11 months of age and was compound heterozygous for a missense c.343T>C p.(Cys115Arg) and a splice variant c.309+5G>A.^3^

Here, we describe 5 individuals, from 3 families with different ethnic backgrounds, presenting with axonal Charcot-Marie-Tooth (CMT) neuropathy and harboring the same homozygous c.309+5G>A splice-site variant. Their chronic, slowly progressive disease predominantly affecting the peripheral nervous system contrasts the previously reported devastating syndromic cases. We set out to functionally characterize this homozygous variant and understand the mechanism underlying the peculiar CMT phenotype of these patients.

## Materials and Methods

### Clinical and electrophysiologic evaluations

Five patients from 3 unrelated pedigrees were diagnosed in 3 hospitals in Spain (University Hospital Marqués de Valdecilla, Santander), Turkey (Dokuz Eylül University Hospital, Izmir), and Greece (Eginition University Hospital, Athens) (family 1, family 2, and family 3, respectively). The Turkish family was included in this study after a review of published literature,^20^ whereas the patients from the Greek pedigree were retrieved via the Solve-RD platform (http://solve-rd.eu/).^21^ Their ethnicity was self-described. All patients underwent an exhaustive clinical assessment including muscle strength testing according to Medical Research Council (MRC) scores. Routine ancillary investigations were done, including brain magnetic resonance imaging (MRI) (T1, T2, and fluid attenuated inversion recovery [FLAIR] sequences) and biochemistry analyses in serum. Needle electromyography (EMG) and nerve conduction studies were performed using standard methods. In the affected patients from family 1, 1H magnetic resonance spectroscopy was performed.

### Exome sequencing (ES) pipeline

ES of the affected individuals from family 1 was performed on genomic DNA using Roche SeqCap EZ Exome Probes v3.0 (Roche Holding AG) for exon capture and NextSeq 150 (Illumina) for paired-end sequencing (150 bp). Sequencing read mapping, variant calling and annotation were done using GenomeComb.^22^ The exomes from the probands of family 2 and family 3 were captured with SureSelect All Exon v7 and v4 kits from Agilent, respectively.^20^^,^^23^ Before this study, potential deleterious variants in neuromuscular diseases genes were excluded in all 3 families, as previously described.^20^^,^^23^^,^^24^ In addition, because of the pronounced movement symptoms observed in the patients from family 3, pathogenic variants in a movement disorders virtual gene panel^25^ were also excluded. In silico and splicing prediction tools were used to predict the pathogenicity of the variants. The prioritized variants were confirmed and segregated in available relatives by Sanger sequencing, as described.^26^

### Homozygosity mapping and haplotype sharing analysis

Exome vcf files were analyzed with the AutoMap software v1.2 to identify shared homozygous regions.^27^ The common shared homozygous regions were queried for single-nucleotide polymorphisms with a minimal coverage of 8 reads and an allele frequency of 70% or less. The constructed genotypes at those loci were compared to establish a putative common haplotype surrounding the *NDUFS6* homozygous variant. Variant dating was performed assuming a correlated genealogy and a span of 20 years per generation, as described previously.^28^

### Cohort screening

A cohort of 665 individuals with CMT (214 demyelinating, 330 axonal, 71 intermediate, and 50 unknown CMT type), and 95 non-5q spinal muscular atrophy (SMA) patients with autosomal recessive inheritance or sporadic cases were screened for genetic variants in exons and exon-intron boundaries of *NDUFS6* (NM_004553.6) using an amplicon target amplification assay.^29^ Sequencing read mapping, variant calling and annotation were done using GenomeComb.^22^ Primers are listed in Supplemental Table 1.

### Lymphoblast cultures establishment and maintenance

Peripheral blood mononuclear cells from the patients and parents of family 1 were isolated and transformed with Epstein-Barr virus (EBV) as described.^30^

### RNA isolation, RT-PCR assays, and cDNA-targeted long-read sequencing

Total RNA was isolated from lymphoblasts using the Universal RNA kit (Roboklon), and the remaining genomic DNA was digested using the Turbo DNA-free kit (Ambio). RNA was transcribed to complementary DNA (cDNA) using the iScript cDNA advanced synthesis kit (Bio-Rad). Targeted long-read sequencing (T-LRS) of *NDUFS6* cDNA was performed using the Flongle flow cell on MinION sequencer (Oxford Nanopore Technologies) with a forward primer in exon 1 and a reverse primer in exon 4, as described.^31^ The generated reads were analyzed using FLAIR v1.5.^32^ Primers are listed in Supplemental Table 1. The protein sequence of the identified splicing isoforms was predicted with the SnapGene software (Insightful Science, available at snapgene.com).

### Mitochondrial fractionation

Patient-derived lymphoblasts were collected by centrifugation at 1500 rpm at 4 °C for 10 minutes and washed with cold phosphate-buffered saline (PBS). The pellets were resuspended in cold mitobuffer (250 mM mannitol, 0.5 mM ethylene glycol tetraacetic acid, and 5 mM 4-(2-hydroxyethyl)piperazine-1-ethane-sulfonic acid, pH 7.4) and lysed by a 26.5G syringe (303800, Becton Dickinson) in 10 strokes. Then, mitochondrial fractions were isolated by differential centrifugation as described.^33^

### Immunoblotting assays

Protein lysates were obtained and transferred to blotting membranes as described.^30^ Membranes were immunoblotted with anti-NDUFS6 (ab195807, 1:1000, Abcam), anti-succinate dehydrogenase complex subunit A (GTX632636, 1:3000, Abcam), anti-α-tubulin (ab14715,1:4000, Abcam), OXPHOS Human WB Antibody cocktail (ab110411, 1:200, Abcam), anti-MTCO2 (ab91317, 1:1000, Abcam), anti-ATP5C1 (60284-1-IG, 1:1000, ThermoFisher Scientific) or anti-VDAC1 (ab14734, 1:1000, Abcam). Results were visualized with chemiluminescence detection (GE Healthcare).

### Flow cytometry analyses

Patient-derived EBV-transformed lymphoblasts were washed twice with prewarmed PBS and incubated for 30 minutes at 37 °C with 20uM tetramethylrhodamine ethyl ester perchlorate (ENZ-52309, Enzo Life Sciences). As positive controls, we incubated cells from healthy individuals with 20 nM carbonyl cyanide-p-trifluoromethoxyphenylhydrazone (ab120081, Abcam) for 5 minutes at 37 °C to depolarize the mitochondrial membrane before staining. After staining, cells were rinsed with prewarmed PBS and analyzed with flow cytometry on MACSQuant Analyzer 10 (Miltenyi Biotec). Median fluorescence intensity was measured using FlowLogic 8.6 software (Inivai Technologies).

### Statistical analysis

GraphPad Prism 9.2.0 was used for statistical analyses.

## Results

### Identification of *NDUFS6* as a CMT gene

**Figure 1:**
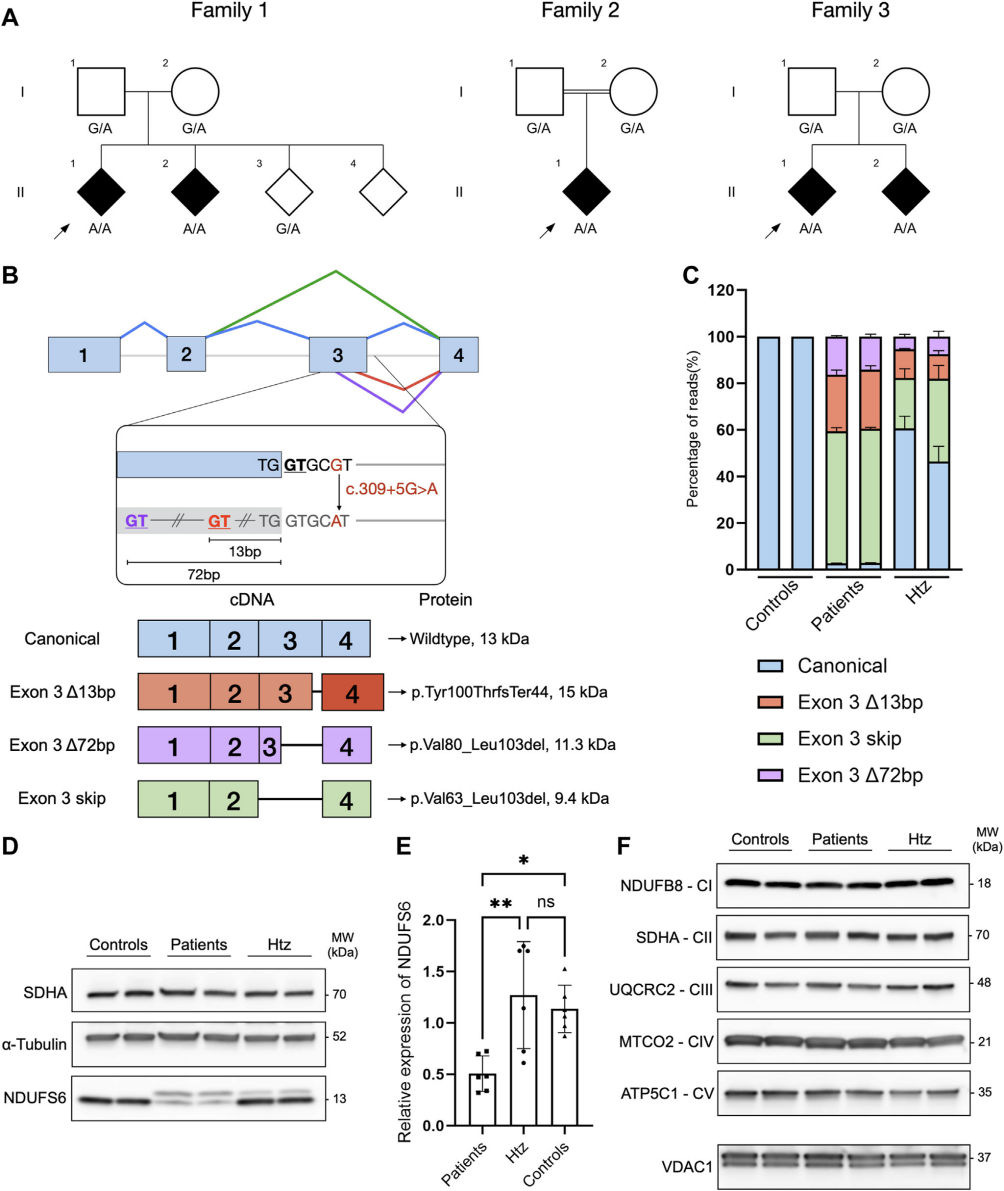
**Genetic and functional analysis of the *NDUFS6* c.309+5G>A splice variant**. A. Segregation analysis of *NDUFS6* c.309+5G>A in families 1-3. Black symbols indicate affected individuals. A double line indicates reported consanguinity. Index patients are indicated with a black arrowhead. B. Top panel: *NDUFS6* (NM_004553.6) is schematically shown with exons 1-4 (blue rectangles) and introns (gray line). Caret-like exon connecting lines depict canonical (blue), exon 3 skip (green), exon 3 Δ72bp (purple), and exon 3 Δ13bp (red) splicing events. The 3’-end of exon 3 and intron 3 are presented in more detail to indicate the location of the *NDUFS6* c.309+5G>A splice variant (red), the canonical splice-donor site (black and underlined) and the cryptic splice-donor sites (red and purple underlined) located at 13 bp and 72 bp upstream of the 3’-end of exon 3, respectively. Bottom panel: schematic representation of the *NDUFS6* transcripts detected in the patients and parents from family 1. The identity of each transcript is indicated on the left, the impact of each splicing on the protein is indicated on the right. C. The relative quantification of *NDUFS6* transcripts sequenced by cDNA T-LRS among the 3 genotypes of interest (heterozygotes, patients, and controls). The chart represents the percentage of reads for each transcript quantified relative to the total amount of reads per sample. The bars indicate the standard error of the mean (*n* = 2). D. Western blot analysis of total protein lysates isolated from lymphoblasts of heterozygotes, patients, and controls with a monoclonal rabbit anti-human NDUFS6 antibody. Monoclonal mouse anti-SDHA and anti-α-tubulin were used as controls for equal loading of mitochondrial and cytosolic fractions, respectively. E. Relative quantification of different NDUFS6 isoforms present in lymphoblasts of heterozygotes, patients, and controls. NDUFS6 band intensity is relative to α-tubulin and normalized to controls. Bar charts are represented as the standard error of the mean (*n* = 3 for each genotype, with 2 biological replicates for each genotype). F. Immunoblotting of mitochondrial fractions from 3 genotypes of interest (heterozygotes, patients, and controls) with monoclonal mouse anti-human NDUFB8, SDHA, UQCR2, and ATP5C1 and monoclonal rabbit anti-human MTCO2 antibodies to target 1 subunit from each OXPHOS complex. Monoclonal mouse anti-human VDAC1 was used as a control for mitochondrial proteins. All statistical analyses were performed using one-way ANOVA. Asterisks denote significance after Tukey’s multiple comparison correction. Abbreviations: Htz, heterozygotes; ∗∗*P* < .01, ∗*P* < .05; ns, not significant; SDHA, succinate dehydrogenase complex subunit A.

We performed ES on 2 Spanish siblings with early-onset axonal CMT. After exclusion of potentially deleterious variants in known CMT genes, the exome was queried for coding non-synonymous variants present in both affected individuals and with an allele frequency below 0.05 in the Genome Aggregation Database (gnomAD)^34^ (Supplemental Table 2). The analysis revealed a homozygous single-nucleotide variant NM_004553.6: c.309+5G>A NC_000005.10: g.1814466G>A in *NDUFS6*. The substitution lies in the splice-donor region of exon 3 (out of 4 exons) and was predicted to cause the loss of the donor site by multiple in silico tools.^35^, ^36^, ^37^ The variant was rare (0.00001193 allele frequency in gnomAD), and no homozygotes were reported.^34^ Sanger sequencing in available relatives showed that the variant co-segregated with the disease (Figure 1A). Notably, the splice-site substitution had already been reported twice in compound heterozygous state to cause Leigh syndrome by Rouzier et al^3^ and Ogawa et al.^16^ Moreover, Rouzier et al^3^ demonstrated that the variant causes exon 3 skipping, and to a lesser degree, allows the residual expression of the canonically spliced *NDUFS6* transcript. Because this gene has never been associated with CMT, *NDUFS6* was deemed a gene of uncertain significance for CMT according to the American College of Medical Genetics and Genomics guidelines.^38^ Therefore, further genetic and functional studies were considered necessary to support the novel genotype-phenotype connection.

Screening of 760 unsolved patients with CMT and non-5q SMA did not yield any additional cases with biallelic *NDUFS6* variants. Queries of Solve-RD database and the literature revealed 3 additional patients from Turkish and Greek families with the same homozygous *NDUFS6* c.309+5 G>A variant (Figure 1A). One of the probands had been reported previously.^20^ Haplotype analysis based on homozygosity mapping using ES data showed that the variant lies on a shared haplotype of 0.74MB on chromosome 5 (Supplemental Figure 2). We estimated that the variant arose 36.8 generation ago (95% CI 4.0-82.1). Assuming an average of 20 years per generation, the most recent common ancestor with the haplotype would have lived 740 years ago (95% CI 80-1640).

### Clinical findings

#### Family 1

**Figure 2:**
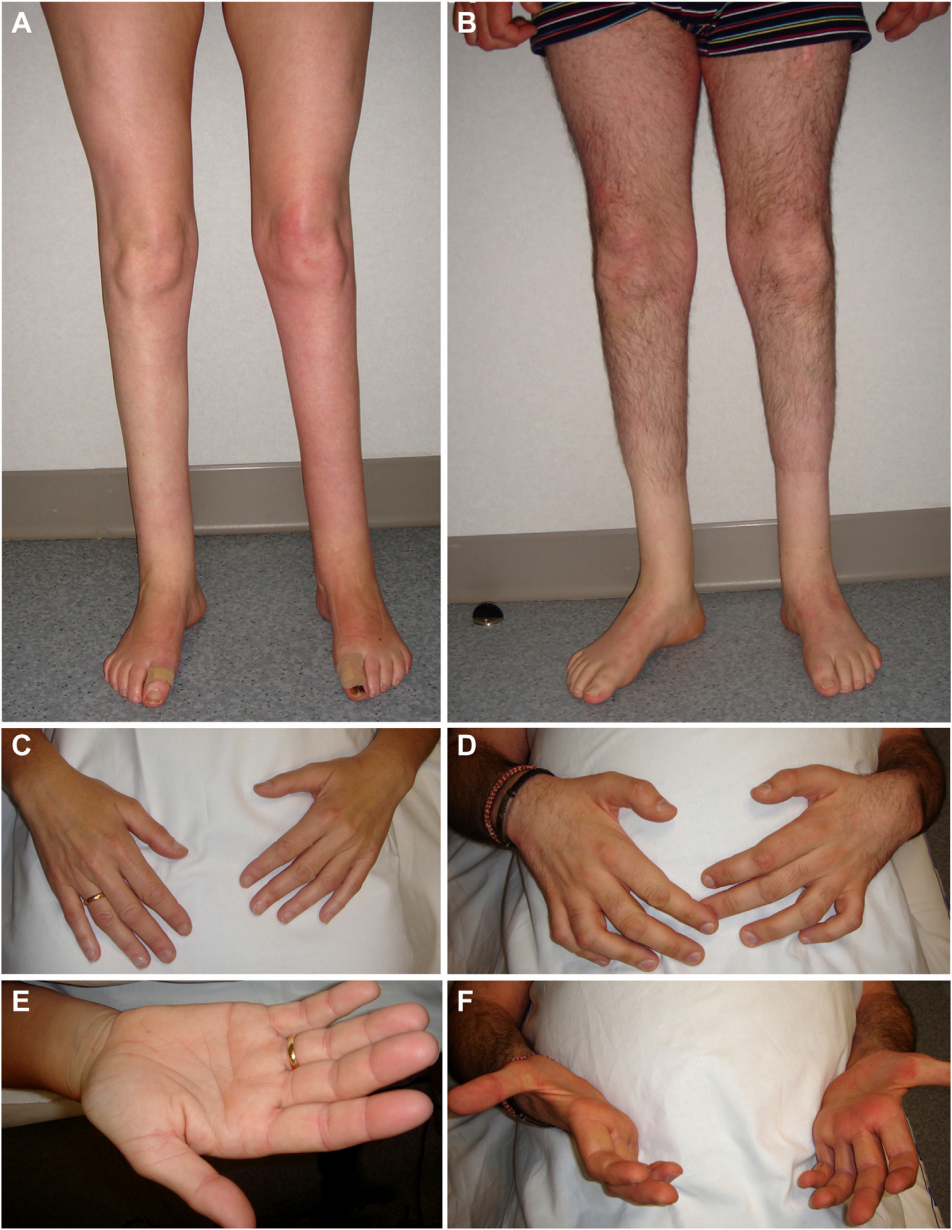
**Clinical features of the patients from family 1.** The proband, II-1, at age 46 years, exhibited marked lower leg amyotrophy (A), and incipient hand wasting involving first dorsal interossei and thenar musculature (C and E). The affected sibling II-2, at age 38 years, shows a more pronounced phenotype, with marked lower leg amyotrophy (B) and advanced wasting of hand musculature with clawing deformity (D, C, and F). Note the absence of proximal lower-limb amyotrophy in both patients (A and B).

The patients experienced delayed walking at 15 months of age, followed by progressive lower-limb amyotrophy and an unsteady gait (Table 1). They also had pes cavus, which required surgical procedures at age 6 years (patient II.2) and ages 10 and 12 years (patient II.1). Over a 30-year period, the disease progressed slowly. In the last evaluation (at ages 52 and 44 years, respectively) the patients showed severe paresis and wasting of foot flexor/extensor muscles (Figure 2A and B) and bilateral steppage gait without support (Supplemental Video 1). Patient II-1 had incipient hand wasting (Figure 2C and E), whereas their sibling had severe hand wasting and weakness (Figure 2D and F). Touch and pain hypoesthesia in a stocking distribution, as well as vibratory hypopallesthesia, were present. Ankle jerks were absent, whereas the remaining tendon reflexes were either normal or brisk. In both patients, there was bilateral gaze-evoked horizontal nystagmus, and neither of them were taking any medications (eg, sedatives, tranquilizers, or anticonvulsants) known to cause this condition.^39^ Clinical evaluation of their unaffected relatives (individuals I-1, I-2, and II-3, Figure 1A) revealed normal findings.

**Table 1:** Clinical characteristics of all patients described in this study who carry the *NDUFS6* c.309+5G>A variant in homozygosity

Clinical features	Family 1		Family 2	Family 3	
	II-1	II-2	II-1	II-1	II-2
Origin	Spanish		Turkish	Greek	
Consanguinity	No		Yes	No	
Clinical diagnosis	Axonal CMT		Axonal CMT	Axonal CMT	Axonal CMT
Age at onset (y)	1.25	1.25	10	10	10
Age at last examination (y)	52	44	19	19	17
Initial symptoms	Late walking		Distal muscle weakness	Unsteady gait, ataxia	Unsteady gait, frequent falls
Main symptoms	Severe distal LL weakness and atrophy, foot deformities		Distal muscle weakness	Severe LL weakness, ataxia	Distal LL weakness
Disease course	Minimal progression	Minimal progression	Minimal progression	Minimal progression	Minimal progression
Gait	Steppage	Steppage	Steppage	Steppage, need bilateral assistance, ankle foot orthoses	Steppage
Muscle weakness (MRC)					
UL proximal	5	5	5	5	5
UL distal	5	0-3	4	4	5-
LL proximal	5	5	5	4	5
LL distal	0	0	Dorsiflexion: 0, plantar flexion: 2	0	Dorsiflexion: 0, plantar flexion: 3
Muscle wasting	Severe dLL, minimal dUL	Severe dLL, dUL	dLL	dLL, minimal dUL	dLL
Tendon reflexes	Achilles: 0, other: 3	Achilles: 0, other: 1	Achilles: 0, knee: 1, other: 2	Achilles: 0, knee: 1, UL: 3	Achilles: 0, other: 2
Skeletal deformities	Pes cavus		Pes cavus	Pes cavus	Pes cavus
Sensory loss					
Light touch	LL	LL	dLL	LL	No
Pain/temperature	LL	LL	dLL	LL	dLL
Proprioception	No	No	No	LL	No
Vibration	LL, dUL	LL, dUL	No	LL, dUL	dLL
Nystagmus	Yes	Yes	Yes	Yes	Yes
Involuntary movements	None	None	None	Tremor, chorea, athetosis	Chorea, myoclonus, left UL dystonia
Brain MRI	Normal	Normal	Normal	“Eye-of-the-tiger” sign in basal ganglia	Unknown
Biochemical assays					
Serum lactate^h^	**2.5 mmol**/**L**^a^	1.9 mmol/L^b^	1.03 mmol/L^c^	2.02 mmol/L^d^	NA
Serum pyruvate^i^	84 μmol/L^e^	85 μmol/L^f^	NA	120 μmol/L^g^	NA
Lactate/pyruvate^j^	**29.8**	22.4	NA	16.83	NA
Additional features	NA	NA	Intellectual disability	NA	Rolandic epilepsy

Abnormal values are highlighted in bold. Muscle weakness scale (MRC): 0 = no contraction, 1 = flicker or trace of contraction, 2 = active movement, with gravity eliminated, 3 = active movement against gravity, 4 = active movement against gravity and resistance, 5 = normal power. Tendon reflexes scale: 0 = absent, 1 = reduced, 2 = normal, 3 = increased, 4 = clonus.

*CMT*, Charcot-Marie-Tooth disease; *dLL*, distal lower limb; *dUL*, distal upper limb; *LL*, lower limb; *UL*, upper limb.

a 22.52 mg/dL.

b 17.12 mg/dL.

c 9.28 mg/dL.

d 18.20 mg/dL.

e 0.74 mg/dL.

f 0.75 mg/dL.

g 1.06 mg/dL.

h Reference range 0.5-2.2 mmol/L (4.5-19.82 mg/dL).

i Reference range 40-130 μmol/L (0.35-1.14 mg/dL).

j Reference value <25.

Serum lactate levels and lactate/pyruvate ratio were minimally elevated in the proband II-1 but normal in patient II-2. Brain MRI and magnetic resonance spectroscopy from the patients showed no abnormalities. Lower-limb nerves were unexcitable in both patients at the nerve conduction studies (Table 2). The proband exhibited normal upper limb compound muscle action potential amplitudes with preserved motor conduction velocity (MCV) and attenuated sensory nerve action potential (SNAP) with minimal slowing of SCV. In individual II-2, distal amplitudes of median and ulnar nerves were unobtainable or severely attenuated, and the velocities were reduced in accordance with the degree of compound muscle action potential attenuation. EMG of the biceps brachii from II-2 indicated an underlying axonal degeneration process (Supplemental Figure 3).

**Table 2:** Electrophysiological studies

NCS		Family 1		Family 2	Family 3	
		II-1	II-2	II-1	II-1	II-2
Median	CMAP	10.8 mV	**0.2 mV**	14.2 mV	11.3 mV	12.1 mV
	MCV	55.2 m/s	**22.5 m/s**	58.4 m/s	58.8 m/s	56.7 m/s
	SNAP	**0.9 μV**	ND	ND	ND	ND
	SCV	**39.7 m/s**	ND	ND	ND	ND
Ulnar	CMAP	14.1 mV	**0.4 mV**	9.3 mV	6.2 mV	5.9 mV
	MCV	62.4 m/s	**39.5 m/s**	51.4 m/s	59.5 m/s	63.5 m/s
	SNAP	**0.5 μV**	ND	**0.3 μV**	ND	ND
	SCV	41.0 m/s	ND	**35.9 m/s**	ND	ND
Peroneal	CMAP	ND	ND	ND	R: ND, **L: 0,19 mV**	**2.1 mV**
	MCV	ND	ND	ND	ND	57.5 m/s
Tibial	CMAP	ND	ND	ND	ND	ND
	MCV	ND	ND	ND	ND	ND
Sural	SNAP	ND	ND	ND	ND	ND
	SCV	ND	ND	ND	ND	ND
EMG						
Tibialis anterior		Neurogenic	Neurogenic	Neurogenic	Neurogenic	Neurogenic
Electrophysiological diagnosis		Axonal CMT	Axonal CMT	Axonal CMT	Axonal CMT	Axonal CMT

Abnormal values are highlighted in bold.

*CMAP*, compound muscle action potential; *EMG*, electromyography; *L*, left; *MCV*, motor conduction velocity; *ND*, not detectable; *NCS*, nerve conduction studies; *R*, right; *SCV*, sensory conduction velocity; *SNAP*, sensory nerve action potential.

#### Family 2

The proband (II-1, Figure 1A) showed gait difficulties and frequent falls from age 10 years because of slowly progressive distal lower limbs weakness. During the last clinical evaluation at 19 years of age, the patient exhibited distal leg amyotrophy with pes cavus, severe paresis of foot flexor/extensor muscles, and mild hand weakness. The patient showed steppage gait but was able to walk independently. Ankle reflexes were absent and patellar reflexes were reduced. There was stocking touch and pain hypoesthesia in distal lower limbs, as well as sensory ataxia. Nystagmus was observed and the coordination exam was normal. In addition, the patient records reported mild intellectual disability. However, it was not possible to recontact the patient to investigate this further.

Serum lactate was within normal ranges and MRI exhibited no abnormal findings. Lower limbs MCVs and SCVs were not detected. Upper limbs MCVs were conserved and single-nucleotide variant were undetected except for the ulnar nerve, which showed significant attenuation of SNAP and mildly reduced SCV.

#### Family 3

The patients from the third pedigree (II-1 and II-2, Figure 1A) began with unsteady gait and frequent falls from 10 years of age. At disease onset, individual II-1 presented with pes cavus, and choreoathetotic and dystonic movements of the upper limbs. From age 10 years, patient II-2 developed temporarily Rolandic epilepsy and from age 13 years they started exhibiting choreoathetotic and dystonic movements of the left upper limb. On the latest neurological examination at ages 19 and 17 years, respectively, there was lower leg amyotrophy with bilateral steppage gait and severe paresis of foot flexor/extensor muscles and minimal hand weakness in both patients. Ankle reflexes were absent, whereas the remaining tendon reflexes were normal or brisk. There was global stocking hypoesthesia and distal lower-limb hypopallesthesia. There was mild gaze-evoked nystagmus, saccadic pursuit, mild kinetic dysmetria, and jerky tremor or jerky chorea (Supplemental Video 2). Neither parkinsonism nor cognitive decline was noted. As in the patients from the other 2 families, there was minimal disease progression. Examination of the parents (individuals I-1 and I-2; Figure 1A) gave normal results.

MRI was performed in individual II-1 at age 12 by their attending pediatric neurologist, who noted a zone of increased signal in globi pallidi, surrounded by a zone of low signal, as per early “eye-of-the-tiger sign.” MRI images were not available for review. Routine laboratory investigations were normal. In both patients, motor and sensory lower-limb nerves were unexcitable except for the peroneal nerves. In upper limbs, MCVs were preserved, whereas SNAPs were unobtainable. EMG of tibialis anterior showed no voluntary activity and active denervation potentials.

#### NDUFS6 splicing analysis

To test that the aberrant splicing events described by Rouzier et al^3^ were also occurring in our patients homozygous for the c.309+5G>A variant, we amplified *NDUFS6* cDNA of EBV-transformed lymphoblasts from the 2 patients from family 1 and their heterozygote parents. T-LRS of *NDUFS6* (NM_004553.6) cDNA led to the identification of 4 *NDUFS6* transcripts expressed by both patients and parents (Figure 1B): (1) wild-type canonical; (2) a transcript missing the last 13 bp of exon 3, r.298_310del p.Tyr100ThrfsTer44, referred as “exon 3 Δ13bp”; (3) a transcript missing the last 72 bp of exon 3, r.239_310del p.Val80_Leu103del, referred as “exon 3 Δ72bp”; and (4) a transcript skipping exon 3, r.188_310del p.Val63_Leu103del, referred to as “exon 3 skip.” Relative quantification of the different *NDUFS6* transcripts is shown in Figure 1C. Patients showed a loss of the canonical *NDUFS6* transcript, with only ∼2.8% expression compared with controls, who exclusively expressed this transcript. The majority of *NDUFS6* cDNA in patients consisted of mutant transcripts (exon 3 skip: ∼57.1%, exon 3 Δ13bp: ∼24.9%, and exon 3 Δ72bp: ∼18%). As expected, approximately 50% of the cDNA of the parents corresponded to the canonically spliced one. The heterozygote parents also expressed mutant transcripts but in a smaller proportion than in the patients (exon 3 skip: ∼28.6%, exon 3 Δ13bp: ∼11.5%, and exon 3 Δ72bp: ∼5.4%).

### Protein expression quantification

The exon 3 skip and Δ72bp *NDUFS6* transcripts were predicted to maintain the open reading frame and encode 2 isoforms of 9.4 and 11.3 kDa in size, respectively. In contrast, the Δ13bp was expected to cause a frameshift and delayed stop codon, resulting in a 15 kDa isoform. Immunoblotting was performed to assess the impact of the c.309+5G>A variant on NDUFS6 protein levels in patient’s lymphoblasts (Figure 1D). One NDUFS6 isoform of the expected normal size (13 kDa) was observed in the parents and the control individuals. In the patients, an isoform of approximately the same size was also present, but in a much smaller proportion. In addition, a novel larger NDUFS6 isoform was discernible in the heterozygote parents, and to a greater extent, in the probands. Signal quantification confirmed a significant loss of total NDUFS6 protein in patients compared with the parents and controls, even when considering both isoforms in the analysis (Figure 1E).

Loss of NDUFS6 has been shown to affect CI assembly and stability.^40^, ^41^, ^42^, ^43^ In turn, CI defects might also disturb the formation of mitochondrial supercomplexes, such as the one composed by CI, Complex III, and Complex IV (CI+III_2_+IV).^1^^,^^44^ Therefore, different subunits of each complex were immunoblotted (Figure 1F) to assess whether the partial loss of NDUFS6 protein affects the stability of CI or more OXPHOS complexes. The experiment showed that CI subunit NDUFB8, which is part of the initial CI subassemblies, was not disturbed by the loss of NDUFS6. Similarly, representative subunits of the rest of the OXPHOS complexes were not affected. Accordingly, flow cytometry analysis with tetramethylrhodamine ethyl ester perchlorate, a dye that accumulates in cells with a hyperpolarized mitochondrial membrane, did not show any alterations in patient-derived cells, indicating that the membrane potential is preserved (Supplemental Figure 4).

### Genotype-phenotype correlations

**Figure 3:**
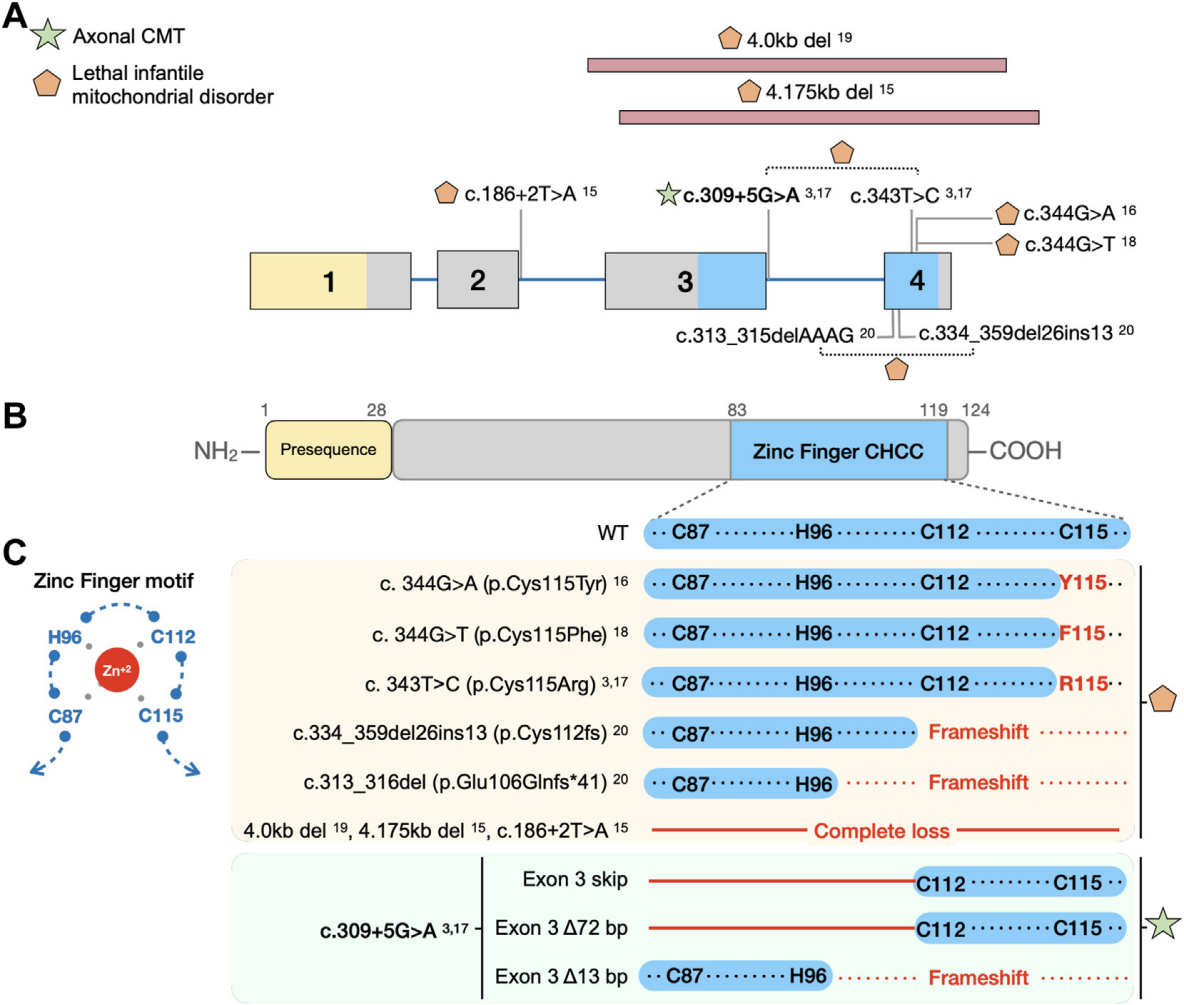
**Clinical and molecular effect of published *NDUFS6* variants.** A. Gene structure of *NDUFS6* (NM_004553.6) with exons 1 to 4 and introns (blue line). The exons are colored according to the protein domain they encode. The location of the pathogenic variants reported in the literature is indicated on the diagram and their superscripts correspond to their number on the reference list. Symbols represent their associated clinical phenotype, as described in the legend on the left top of the figure. A dotted line in between 2 variants indicates reported compound heterozygosity. The pink elongated rectangle indicates a deletion. B. The protein domains of NDUFS6. (C) Left panel: Visual representation of the zinc-finger motif (zf-CHCC). The bound Zinc atom is represented as a red circle. Right panel: Simplified amino acid sequence of the region corresponding to the zinc-finger motif. Below the sequence of wild-type NDUFS6, the previously reported NDUFS6 variants and their consequence on the motif are shown and shaded in yellow. The effect of the aberrantly spliced transcripts found in the patients carrying the c.309+5G>A variant is shaded in green. Red residues indicate an altered amino acid sequence. Red lines indicate regions lost due to the variants. Symbols represent their associated clinical phenotype, as described in the legend on the top left panel.

Next, we performed a systematic analysis of all published pathogenic *NDUFS6* variants and their associated phenotype to understand the potential mechanisms underlying the wide phenotypic spectrum in these patients (Figure 3A). There are 9 pathogenic recessive variants in the literature,^3^^,^^14^, ^15^, ^16^, ^17^, ^18^, ^19^ and all of them lead to disruption of a functional zinc-finger domain in the C-terminal region of NDUFS6. This domain contains a CX_*8*_*-*_*9*_*HX*_*14*_*-*_*15*_*CX*_*2*_*C* motif with 3 cysteines and 1 histidine (zf-CHCC, C87-H96-C112-C115) that coordinate the Zn^2+^-atom at the interface of 2 functional modules of CI (Figure 3C, left panel). Notably, this domain has been demonstrated to play an essential role in CI assembly, stability, and function.^2^, ^3^, ^4^, ^5^^,^^40^ Studies in *NUMM*, *NDUFS6*’s ortholog in *Yarrowia lypolytica*, revealed that loss of the C115 residue had the most deleterious effect on CI assembly and enzymatic activity, whereas disruption of the other components of the zinc-finger domain (corresponding to the human C87, H96, and C112) were better tolerated.^4^ Consistently, the reported deletions or splice-site variants cause protein truncation prompting the complete loss of the domain, whereas the missense variants published cause the substitution of the residue C115 within the domain (Figure 3C, right panel). The patients carrying these variants presented with an early-onset rapidly progressive lethal mitochondrial syndrome. Contrarily, the cDNA analysis of our index patients showed that most (∼80%) (Figure 1C) of the aberrantly spliced *NDUFS6* transcripts retain exon 4 and its reading frame. The resulting isoforms have a partial deletion of the zinc-finger domain; yet, they maintain the critical C115 residue. Therefore, their milder phenotype could be attributed to the expression of these partially functional mutant isoforms with a preserved C115 residue.

## Discussion

We identified 5 patients from 3 families presenting with slowly progressive axonal peripheral neuropathy as their predominant symptom and carrying a homozygous *NDUFS6* c.309+5G>A variant. The same genetic alteration has previously been associated with severe Leigh syndrome; therefore, it was not initially identified or interpreted as pathogenic for CMT using the conventional prioritization algorithms. However, follow-up genetic and functional studies demonstrated that this is a founder variant leading to a partial loss of NDUFS6 function at both mRNA and protein level. Based on these findings, this variant can now be classified as pathogenic for peripheral neuropathy (Supplemental Table 3), and *NDUFS6* should be considered as a novel gene for axonal CMT.

Peripheral neuropathy is a common symptom of mitochondrial disorders, occurring in approximately one-third of the patients.^45^, ^46^, ^47^ However, it is typically part of a syndromic phenotype together with other neurological and extra-neurological manifestations, such as encephalopathy, myopathy, cardiac disease, and/or renal dysfunction.^45^^,^^47^ Remarkably, peripheral neuropathy is the main clinical feature in our patients. In such rare cases, it is possible to overlook the mitochondrial etiology because of the absence of symptoms in other body organs or systems.^48^ Our findings on *NDUFS6*, together with similar observations on other OXPHOS genes (eg, *SURF1*^49^ and *SCO2*^50^), suggest that seemingly distinct clinical entities such as CMT and mitochondrial disorders overlap in their genetic etiology. Therefore, we underscore the importance of testing genes associated with primary mitochondrial disorders in patients with CMT or other neuromuscular diseases without multisystem involvement.

In addition to the chronic peripheral neuropathy, all patients presented with gaze-evoked nystagmus. Nystagmus has rarely been associated with CMT.^51^^,^^52^ Yet, it is a common manifestation of OXPHOS disorders due to CI defects.^53^^,^^54^ Because medication-induced nystagmus was excluded in our patients, it is possible that NDUFS6 defects cause dysfunction of neural structures involved in repetitive eye movements including the vestibulocerebellum or its connections with the brainstem.^39^

It is worth noting the intra- and interfamilial phenotypic variability observed among individuals carrying the same pathogenic variant. The siblings from family 1 show differences in CMT severity, with individual II-2 exhibiting more pronounced upper limb symptoms. In contrast to family 1, the patients from family 2 and family 3 show additional features indicative of central nervous system (CNS) involvement, ranging from ataxia, tremor, and choreoathetosis (family 3) to mild intellectual disability (family 2). This variability was also reflected on the MRI and biochemical results. The exact reasons underlying the observed phenotypic variability are not clear. Because pathogenic variants in genes previously associated with neurological disorders were excluded, the observed clinical spectrum could reflect individual differences in nuclear or mitochondrial genetic background or environmental modifiers.^55^

Strikingly, the *NDUFS6* c.309+5G>A variant was previously reported to cause Leigh syndrome associated with death in the first year of life.^3^ It was remarkable to find the same variant in 5 patients (3 adolescents and 2 adults), the eldest being 52 years old. Expression studies in patient-derived lymphoblasts suggest that the reason for the unexpectedly mild clinical phenotype are alternative splicing events resulting in a partial loss of NDUFS6 function. Using cDNA T-LRS, we discovered that next to the exon 3 skip transcript described by Rouzier et al,^3^ the loss of the splice-donor site also causes the activation of cryptic splice-donor sites, leading to the expression of 2 additional mutant transcripts lacking fragments of exon 3 (Figure 1B). These results highlight the added value of the novel sequencing technologies for the correct identification and quantification of alternatively spliced transcripts. Moreover, considering that the canonical transcript is expressed at negligible levels (approximately 3%) (Figure 1C), we believe that this canonical transcript is not sufficient to maintain NDUFS6 function, as previously suggested.^3^ Likewise, protein expression analysis revealed a significant loss of NDUFS6 protein in patients with presence of at least 2 mutant isoforms. We reckon that the observed NDUFS6 isoforms correspond to the aberrantly spliced transcripts and are likely the ones that partially compensate for the loss of wild-type NDUFS6. Additional experiments are required to distinguish specifically which mutant transcripts give rise to the detected protein isoforms. Similar examples in which aberrant splicing attenuates a trait have been described in Leigh syndrome because of pathogenic variants in *NDUFS3* and *NDUFAF6*,^56^ in xeroderma pigmentosum-Cockayne syndrome complex^57^ and in breast cancer risk due to variants in *BRCA2*.^58^

Systematic analysis of all published *NDUFS6* patients allowed us to establish important genotype-phenotype correlations. NDUFS6 has a functional zinc-finger domain that is essential for CI assembly and stability,^2^, ^3^, ^4^, ^5^^,^^14^ in which the C115 residue plays the most essential role.^4^ All reported *NDUFS6* patients with the lethal mitochondrial phenotype lack the entire functional domain or this C115 residue (Figure 3C).^3^^,^^14^, ^15^, ^16^, ^17^, ^18^, ^19^ Contrarily, our patients express 2 aberrantly spliced *NDUFS6* transcripts with a disrupted zinc-finger domain but an intact C115 residue. These findings highlight the importance of the integrity of the last cysteine (C115) of the zinc-finger domain for NDUFS6 functionality because it might differentiate between a lethal and viable phenotype.

Previous studies show that loss of NDUFS6 halts the final step of CI assembly and leads to the accumulation of a CI intermediate.^40^, ^41^, ^42^, ^43^ Consistently, we did not detect changes in the early assembled subunit NDUFB8 in mitochondria isolated from lymphoblastoid cells of the patients from family 1. Furthermore, no defects in the mitochondrial membrane potential were observed in patient-derived cells. Thus, we reckon that the expression of aberrantly spliced isoforms might mitigate the potential metabolic consequences of the complete loss of canonical protein. In addition, such a mild deficiency might be undetectable depending on the tissue or cells used, as seen previously.^3^^,^^56^ In agreement with this, the patient with fatal Leigh syndrome carrying the *NDUFS6* c.309+5G>A and the c.343 T>C (p.Cys115Arg) variants showed normal CI activity in fibroblasts and muscle in spite of the observed assembly and stability defect of CI.^3^ Further functional studies are needed to elucidate the impact of the splice variant in the function and assembly of CI.

In summary, we report that *NDUFS6* is a novel disease-causing gene for axonal CMT, expanding the clinical spectrum of *NDUFS6*-related mitochondrial disorders. Our results emphasize the clinical and pathophysiologic overlap between CMT and mitochondrial disorders. Based on our findings, it is advisable to screen genes associated with primary mitochondrial disorders in patients with CMT, with or without additional CNS features. Moreover, this work highlights the critical role that alternative splicing plays in the modulation of the severity of a genetic disorder. Therefore, special consideration should be dedicated to interpreting splice variants and their potential impact on a disease's prognosis.

## Data Availability

Experimental data not published within this article can be shared by the corresponding author (albena.jordanova@uantwerpen.be) on request from any qualified investigator.

## Conflict of Interest

The authors declare no conflicts of interest.
