# Supplementary material for: Alternative splicing expands the clinical spectrum of NDUFS6-related mitochondrial disorders
Source: Genet Med. 2024 Jun;26(6):101117. doi: 10.1016/j.gim.2024.101117 (PMC11180951; doi:10.1016/j.gim.2024.101117)
Supplement: Supplementary Material [file mmc3.docx]

## Supplementary Material

**Table S1. PCR amplification of *NDUFS6* cDNA or gDNA.**

| Target | Primers | Thermal cycler protocol | |
| --- | --- | --- | --- |
| Targeted resequencing of *NDUFS6* (NM_004553.6) | | | |
| Exon 1 | Forward: ^5’^AACGGGTGACCACCTGAAT^3’^  Reverse: ^5’^ACAGCACAACCTTACCACGAC^3’^ | 10’ 98°C  45’’ 95°C  25x  45’’ 60°C  2’ 68°C  10’ 72°C | |
| Exon 2 | Forward: ^5’^CCTCCTTAGCTCCACCTTCC^3’^  Reverse: ^5’^GAACTGTTAGGGTGCTTGCAATA^3’^ | |  |
| Exon 3 | Forward: ^5’^ATGTGTGTGTAGGCCCTGAAT^3’^  Reverse: ^5’^GCAGGTAGAGAAGAGGACAGAAA^3’^ | |  |
| Exon 4 | Forward: ^5’^TCAGCCTGGAACCGAGAT^3’^  Reverse: ^5’^TCACTTCCAAAGCCCAGACTA^3’^ | |  |
| Primers were supplemented with prefixes:  Forward: ^5’^TCGTCGGCAGCGTCAGATGTGTATAAGAGACAG^3’^  Reverse: ^5’^GTCTCGTGGGCTCGGAGATGTGTATAAGAGACAG^3’^ | | |  |
| Targeted cDNA long-read sequencing of *NDUFS6* (NM_004553.6) | | | |
| NDUFS6 cDNA | Forward: ^5’^TTTCTGTTGGTGCTGATATTGCGGCGGCGATGACCTTCTG^3’^  Reverse: ^5’^ACTTGCCTGTCGCTCTATCTTCCTAGTGGTGGTGCTGTCTGA^3’^ | | 2’ 95°C  30’’ 95°C  35x  1.15’ 68°C  5’ 68°C |
| Primers were supplemented with prefixes:  Forward primer: ^5’^TTTCTGTTGGTGCTGATATTGC^3’^  Reverse primer: ^5’^ACTTGCCTGTCGCTCTATCTTC^3’^ | | |  |

**Table S2. Exome sequencing quality, results and filtering strategy.**

| **WES statistics** | **Family 1 II-1** | **Family 1 II-2** |
| --- | --- | --- |
| Fraction on target | 78.1% | 78.2% |
| Average sequencing depth on target | 85.4% | 88.8% |
| Coverage of target region | 96.6% | 96.8% |
| Fraction of target covered with at least 20x | 91.2% | 91.8% |
| Total number of variants | 173,228 | 174,000 |
| **Virtual CMT panel filtering** | Negative | |
| **Exome-wide filtering strategy** | Number of variants | |
| Homozygous variants | 51,126 | |
| Shared variants | 38,483 | |
| In coding or splice regions | 6,830 | |
| <0.05 MAF in all gnomAD and in non-Finish Europeans | 58 | |
| No homozygotes in gnomAD | 52 | |
| Non-synonymous | 35 | |
| IGV inspection | 4 | |
| Co-segregation in the family | 1 | |

Abbreviations: MAF: Minor allele frequency, IGV: Integrative Genomics Viewer.

**Figure S1. Amino acid sequence conservation of NDUFS6 zinc finger domain in different species.** The key residues of the zinc finger domain (zf-CHCC) are colored in blue.

**
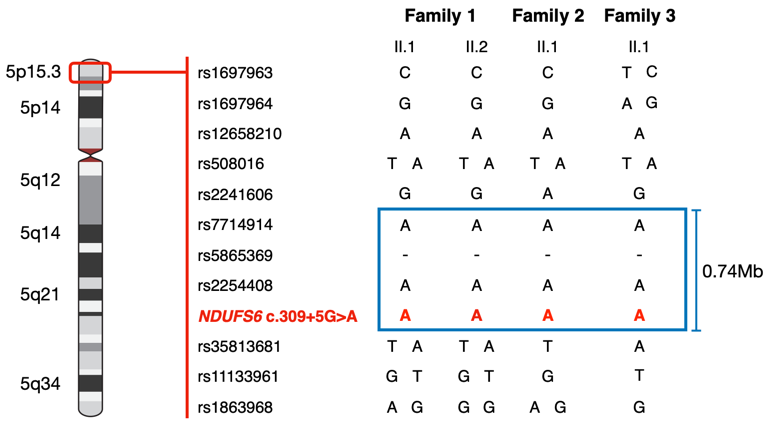
Figure S2. Haplotype sharing analysis from ES data.** The haplotype surrounding the *NDUFS6* c.309+G>A variant (depicted in red) is shown. The haplotype shared by the patients is depicted by the blue box.


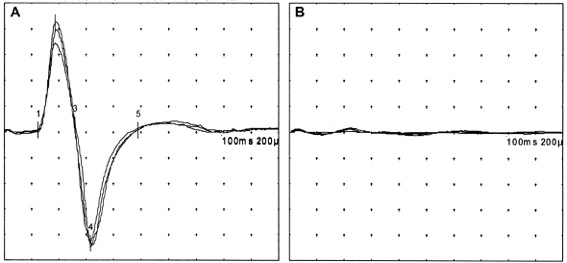
**Figure S3. T-reflex recordings of proband II-2 of family 1.** (A) Biceps T-reflex is preserved both in latency and morphology of T-wave responses; these findings are compatible with a primary axonal process. (B) Achilles T-reflex is absent as expected based on the absent ankle jerk and lower-limb nerve inexcitability.

**
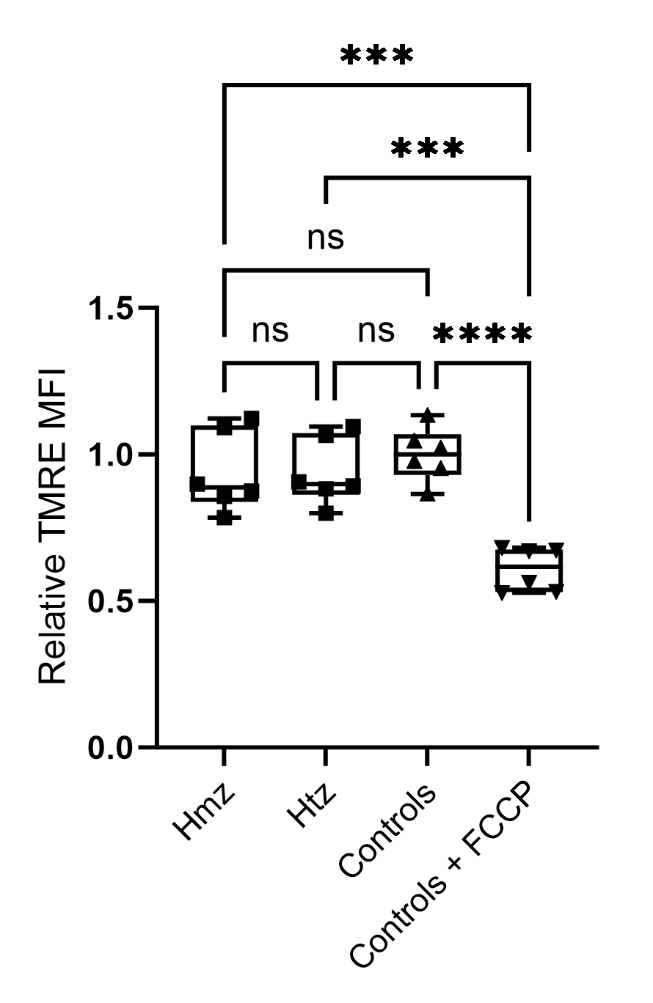
**

**Figure S4. Flow cytometry analysis to estimate mitochondrial membrane potential based on TMRE relative median fluorescence intensity normalized to controls.** Control cells treated with FCCP, which depolarizes the mitochondrial membrane, were used as positive control. Data is shown in a boxplot where the central line represents the median, the box limits represent the upper and lower quartiles and the whiskers indicate the minimum and maximum values (n = 3 for each genotype, with 2 biological replicates for each genotype). Statistical analyses were performed using one-way ANOVA. Asterisks denote significance after Tukey’s multiple comparison correction. Abbreviations: Htz: heterozygotes, ****P<0.0001, ***P<0.001, **P<0.01, *P<0.05, ns = not significant.

**Table S3. Variant classification according to the guidelines of the American College of Medical Genetics (ACMG)^1^**

| **NDUFS6 variant: NC_000005.10(NM_004553.6): c.309+5G>A** | | |
| --- | --- | --- |
| **Category** | **Criteria** | **Reason** |
| **PM2** | Absent from controls (or at extremely low frequency if recessive) | 3/251,404 alleles (allele frequency 0.00001193) in gnomAD v2.1.1 |
| **PP1** | Cosegregation with disease in multiple affected family members in a gene definitively known to cause the disease | The variant segregated with the disease in all three families. |
| **PS3** | Well-established in vitro or in vivo functional studies supportive of a damaging effect on the gene or gene product | Splicing analysis and immunoblotting performed in this study. Previous functional studies in Rouzier et al.^2^ |
| **PP3** | Multiple lines of computational evidence support a deleterious effect on the gene or gene product | CADD^3^ 22.7.  MaxEntScan^4^, NNSPLICE^5^, SSF-like^6^ predict ~70.6% chance for loss of splice donor site of exon 3 |
| **PM4** | Protein length changes as a result of in-frame deletions/insertions in a nonrepeat region or stop-loss variants | Splice variant leads to expression of multiple isoforms with variable in-frame deletions of exon 3. |
| **1 Strong (PS3) + 2 moderate (PM2, PM4) + 2 supporting (PP1, PP3)** | **Classification: Pathogenic** | |

**Legends of supplementary videos**

**Video S1. Gait impairment in patients from family 1.** Patients II-1 and II-2 present steppage gait and are unable to tip toe or heel walk.

**Video S2. Involuntary movements in patients from family 3.** Patient II-1 shows a jerky action tremor of the upper limbs, predominantly in the left upper limb. Patient II-2 presents choreoathetoid movements in distal upper limbs, which are more pronounced in the upper left limb.

**References**

1. Richards S, Aziz N, Bale S, et al. Standards and guidelines for the interpretation of sequence variants: a joint consensus recommendation of the American College of Medical Genetics and Genomics and the Association for Molecular Pathology. *Genetics in Medicine.* 2015;17(5):405-423.

2. Rouzier C, Chaussenot A, Fragaki K, et al. NDUFS6 related Leigh syndrome: a case report and review of the literature. *J Hum Genet.* 2019;64(7):637-645.

3. Kircher M, Witten DM, Jain P, O'Roak BJ, Cooper GM, Shendure J. A general framework for estimating the relative pathogenicity of human genetic variants. *Nat Genet.* 2014;46(3):310-315.

4. Yeo G, Burge CB. Maximum entropy modeling of short sequence motifs with applications to RNA splicing signals. *J Comput Biol.* 2004;11(2-3):377-394.

5. Reese MG, Eeckman FH, Kulp D, Haussler D. Improved splice site detection in Genie. *J Comput Biol.* 1997;4(3):311-323.

6. Shapiro MB, Senapathy P. RNA splice junctions of different classes of eukaryotes: sequence statistics and functional implications in gene expression. *Nucleic Acids Res.* 1987;15(17):7155-7174.
